# Investigation of Strength Characteristics and Microstructure of Cemented Tailings Backfill

**DOI:** 10.3390/ma19091816

**Published:** 2026-04-29

**Authors:** Zongwen Wang, Huan Zhang, Xiaofeng Li, Shihu Shi, Biyao Geng, Zhenjiang Wen, Hengtao Wang

**Affiliations:** 1 Zhaojin Mining Industry Co., Ltd., Zhaoyuan 265499, China; xdjkwzw@163.com; 2 China ENFI Engineering Corporation, Beijing 100038, China; shish@enfi.com.cn (S.S.); gengby@enfi.com.cn (B.G.); wenzj@enfi.com.cn (Z.W.);; 3 Key Laboratory of Safe and Green Mining of Metal Mines with Cemented Paste Backfill National Mine Safety Administration, Beijing 100038, China; 4 Inner Mongolia Velasco Mining Co., Ltd., Chifeng 025372, China

**Keywords:** tailings, uniaxial compressive strength, fluidity, microstructure

## Abstract

The strength of cemented tailings backfill (CTB) is influenced by multiple factors, with the type of cementitious material playing a crucial role in determining the strength of the backfill. To investigate the influence of two different cementitious materials on the strength of CTB, based on the fundamental physicochemical properties of tailings, flow characteristic tests and uniaxial compressive strength (UCS) tests of the backfill were conducted using a cementitious backfill slurry prepared from tailings. Representative proportioned backfill specimens were selected for X-ray diffraction (XRD) and scanning electron microscope (SEM) microstructural analysis to study the evolution patterns of backfill strength influenced by cementitious material type, cement-to-tailing (c/t) ratio, and slurry concentration. The results indicate that the tailings exhibit favorable gradation but unstable continuity. Furthermore, a high content of clay minerals such as kaolinite, along with the presence of fluorine (F) and phosphorus (P), adversely affects the strength of fillers. In terms of slurry flowability, the fluidity of cementation powder filling slurry is generally superior to that of cemented filling slurry. In identical conditions, the strength of cementation powder fillers at all ages is significantly higher than that of cement. As the c/t ratio decreases, the strength advantage of cementation powder fillers becomes even more prominent. Compared to cemented fillers, the hydrated product calcium silicate hydrate gel (C-S-H gel) in cementation powder fillers is more abundant, while the hydrated product calcium aluminate hydrate (CAH) is coarser. This microscopic structural difference explains the strength characteristics of the fillers.

## 1. Introduction

In cemented paste backfill, the determination of cement dosage primarily depends on the balance between strength requirements and economic costs. From a strength perspective, different mining methods demand varying unconfined compressive strengths of the backfill (typically ranging from 0.5 to 5 MPa); the higher the strength requirement, the greater the cement dosage required. From a cost perspective, as cement constitutes a major component of backfill costs, its dosage should be optimized to the greatest extent possible while meeting strength and transportability requirements, thereby reducing backfill operation costs and minimizing carbon emissions. In addition, factors such as particle size distribution and the content of harmful impurities in tailings can affect cement hydration efficiency, indirectly influencing the determination of cement dosage.

With the depletion of surface mineral resources, mining operations are gradually shifting to underground extraction [1,2,3,4]. To ensure the safe and stable operation of underground mining, engineering practice typically requires the strength of the fillers to maintain a high level of safety margin [5,6,7,8]. Currently, this goal is generally achieved by increasing the cement content; however, this approach directly leads to a substantial increase in the amount of cementitious materials, significantly raising the backfilling cost and seriously affecting the economic efficiency of the mine [9,10,11]. Therefore, it has become a key issue in the field of backfill mining technology to deeply understand the mechanisms of strength formation and evolution in backfills, and to develop methods to enhance strength beyond just the use of cement [12,13,14]. By studying the optimization of backfill material proportions, types of cementitious materials, and the microstructure of the backfill, the strength and economic efficiency of fillers can be improved simultaneously, which is crucial for promoting green mine construction and efficient resource development [15,16].

Currently, researchers have conducted extensive studies on the strength of fillers [16,17,18,19,20,21,22,23]. Ma [24] conducted UCS tests and Brazilian splitting tests on fillers mixed with polypropylene fibers to investigate the effects of different fiber contents on the damage evolution of fillers under underground water erosion. Xiong [25] studied the impact of steel fiber content on the mechanical properties of fillers and observed using digital imaging techniques that steel fibers can effectively restrain the propagation of cracks in fillers, preventing specimen deformation and enhancing the mechanical performance of the filler. Liu [26] used regression analysis to fit the strength of fillers and the content of hydration products, analyzing the strength mechanisms of fillers made with both cement and cementation powder. Jia [27] simulated factors such as in situ backfill pressure and temperature in mining operations to study the effects of curing pressure and temperature on filler strength; curing pressure benefits early strength, while filler strength initially increases and then decreases with rising curing temperature. Zhang [28] explored the mechanical properties of industrial coal slag-activated high-sulfur tailings fillers and conducted orthogonal tests to analyze the effect of backfill material proportions on filler strength. Xue [29], based on Hopkinson bar tests, found that the strength of lithium slag–cement–tailings cemented fillers shows a trend of first increasing and then decreasing under dynamic impact.

In practical mining backfilling, adding fiber materials, activators, and increasing curing temperature are effective methods to improve the mechanical properties of fillers [30,31,32]. However, research on the mechanisms of filler strength enhancement is relatively limited. Therefore, this study uses full tailings from a mine and two types of cementitious materials as raw materials to conduct UCS tests on fillers. By combining XRD and SEM analyses of hydration products and other microstructural characteristics, it investigates the mechanisms by which the type of cementitious material, the c/t ratio, and filling slurry concentration affect filler strength, aiming to provide scientific data reference for tailings filling in metal mines.

## 2. Test Materials and Methods

### 2.1. Materials

To explore the influence of the type of cementitious material on the strength of the backfill, cement and cemented powder were respectively employed as the cementitious materials in the backfilling experiments. The materials used in this test mainly include the whole tailings of a certain fluorite mine, cement, and fly ash. The whole tailings serve as the primary filling material, and their basic physical and chemical properties have an important impact on the properties of the filling slurry. The basic physical parameters of the whole tailings mainly include density, loose density, and porosity. In this experiment, the density and loose density of the whole tailings were measured using the density bottle method SL237-006-1999 and the relative density method SL237-010-1999, respectively [33]. The basic physical parameters of the whole tailings are shown in Table 1.

**Figure 1:**
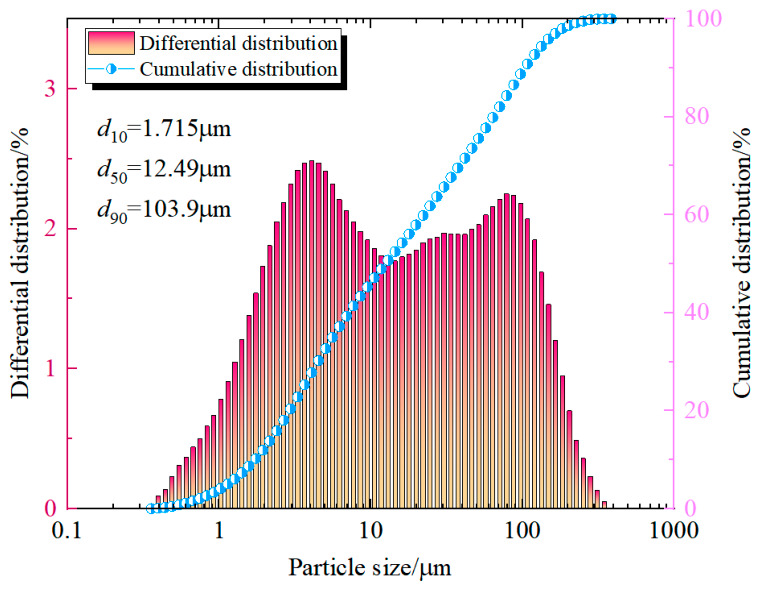
The particle size distribution of the tailings samples.

Measuring the particle size distribution of tailings provides a reference for designing the filling slurry concentration, mix ratio, and other process requirements. The particle size distribution of the whole tailings was tested using an LS-POP (6) laser particle size analyzer (Zhuhai, China) from Malvern Instruments. The particle size distribution curve of the whole tailings is shown in Figure 1. According to the laser particle size test results, over 80% of the fine particles in the fluorite mine whole tailings from Inner Mongolia are below 200 mesh. The particle size characteristic parameters *d*_10_, *d*_30_, *d*_50_, *d*_60_, *d*_90_, and *d*_av_ are 1.72 µm, 4.47 µm, 12.49 µm, 22.48 µm, 103.9 µm, and 35.28 µm, respectively. Using the formulas for the particle size uniformity coefficient *C_u_* and the curvature coefficient *C_c_*, as shown in Equation (1) and Equation (2), respectively, the calculations show *C_u_* = 13.41 > 10 and *C_c_* = 0.57 < 1 for the whole tailings. This does not meet the continuity requirement, indicating a relatively good grading but unstable continuity, with a lack of coarse particles.

The XRF results of materials, measured by X-ray fluorescence spectrometry (Shenzhen, China), are shown in Table 2. The cement is P.O 42.5 cement produced by Anhui Conch Cement Co., Ltd. (Wuhu, China). The cementation powder, a new cementitious material independently developed by China ENFI Engineering Corporation (Beijing, China), is produced by mixing slag, cement clinker, and desulfurization gypsum at a specific ratio. The densities of the cement and the cementation powder are 3.167 g/cm^3^ and 2.955 g/cm^3^, respectively.

(1) $$C_{u}=\frac{d_{60}}{d_{10}}$$

(2) $$C_{c}=\frac{{d_{30}}^{2}}{d_{10}\times d_{60}}$$

### 2.2. Research Methods

#### 2.2.1. Flow Performance Test

(1) Slump and spread test

The slump test is carried out with reference to the concrete test slump experimental method. The standard slump cylinder size used is: bottom diameter 200 mm, top diameter 100 mm, and barrel height 300 mm. Pour and tamp the prepared slurry from the upper mouth of the slump cylinder, scrape the upper mouth flat with a steel ruler, clean the area around the bottom of the barrel, lift the slump cylinder vertically at a uniform speed, and complete the lifting process within about 5~10 s.

(2) Rheological test

The rheological tests of the filling slurry with different ratios were carried out using an RS-SST soft solid rheometer, and rheological parameters such as yield stress and plastic viscosity were obtained. The accompanying Rheo 3000 software (RST-SST Rheometer (7123055)V1.4.3) features automatic testing, automatic data acquisition, data storage, and analysis functions. The instrument has a testing accuracy of ±1%, a maximum torque of 100 mNm, and a rotational speed range of 0.02–1300 rpm. The expression for shear stress is shown in Equation (3).

(3) $$\tau =\tau_{0}+\eta \times \gamma$$

where *τ* is the shear stress, Pa; *τ*_0_ is the yield stress, Pa; *η* is the plastic viscosity, Pa·s; and *γ* is the shear rate, s^−1^.

(3) Bleeding and shrinkage test

Prepare several 500 mL airtight containers, fill the slurry mixture at one time, and then cover the containers to prevent evaporation. Aspirate the bleeding every 20 min with a pipette until there is no bleeding for three consecutive times. Each suctioned volume of water is injected into a stopped graduated cylinder, and finally the total amount of bleeding is calculated.

#### 2.2.2. Strength Performance Test

The filling test block is prepared by filling the test material with tailings, cementitious material and water according to a certain ratio. The ratios of cementitious material and sand to the test block of the filling body are 1:2, 1:3, 1:6, 1:8 and 1:15. The concentrations are 64%, 66%, 68% and 70%; the curing ages were 3 days, 7 days, and 28 days. In this experiment, test blocks with a length × width × height of 70.7 mm × 70.7 mm × 70.7 mm were prepared by the casting mold test method. After the specimen is poured, it is placed in the HBY-40A constant temperature and humidity standard curing box for maintenance. After the curing time, the WHY-300/10 microcomputer control pressure testing machine was used to test the strength and performance of the filling body test block. In order to improve the accuracy of the test data and avoid random errors, three specimens were tested in each group, and the average value was adopted.

#### 2.2.3. Microstructure Testing

To investigate the effects and mechanisms of two cementitious materials on filling body strength, the phase composition and hydration products were characterized by XRD and SEM at the designated curing ages. When preparing the sample, the sample is soaked in anhydrous ethanol, the sample hydration is stopped, and after drying, part of the sample is ground into powder for XRD testing, and the other part of the sample is made into a square test block with a side length of 10 mm for SEM testing.

(1) XRD Test. Before the test, the test samples were pretreated and ground into powder form, and the mineral composition of the whole tailings was qualitatively analyzed by an X-ray diffraction analyzer.
(2) SEM Test. After carbon spraying of the filling test sample, the sample was put into the electron microscopy scanning equipment for microscopic testing and analysis.

## 3. Analysis of Filler Flowability Results

### 3.1. Analysis of Slump and Spread Results

**Figure 2:**
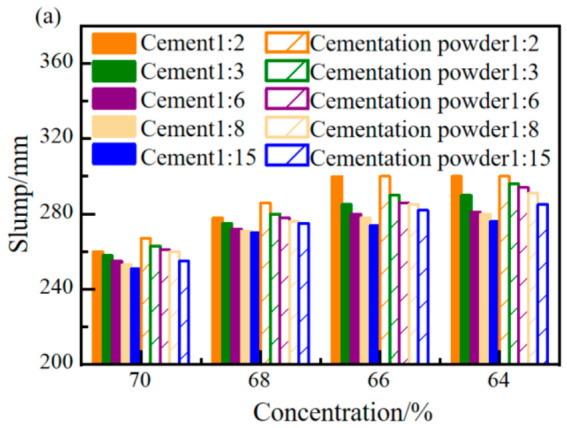

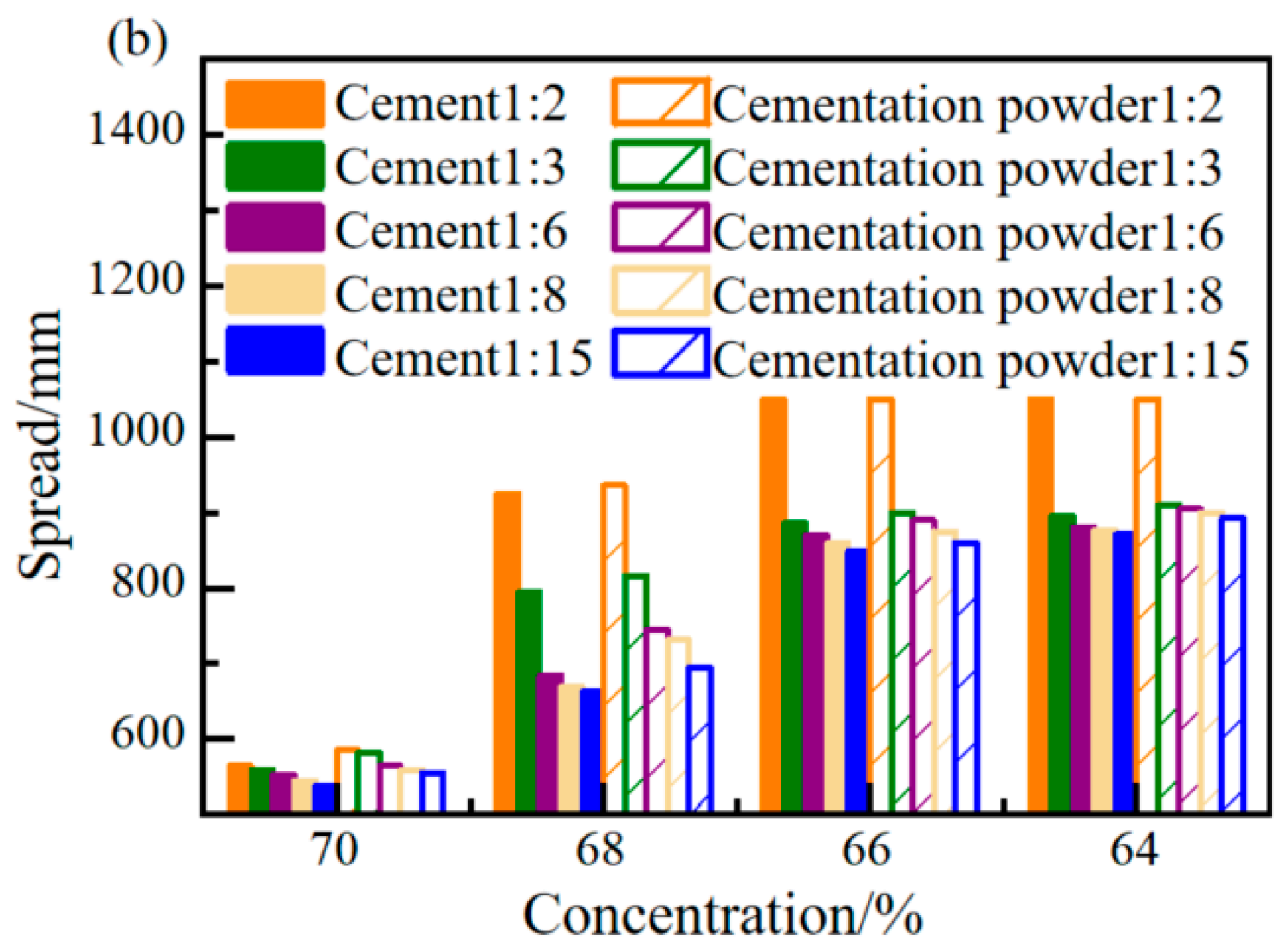
Test results for slump and spread of two cementitious materials: (**a**) slump; (**b**) spread.

The changes in slump and spread of filling slurries made from two types of cementitious materials under different mix conditions were analyzed. The results are shown in Figure 2. According to the experimental results, it can be seen that, when comparing slurries with cement and cementation powder as the cementitious materials, under the same conditions, both the slump and spread of the cementation powder are greater than those of cement, indicating that under the same conditions, the flowability of the slurry made with cementation powder is better than that with cement.

The slump and spread of both types of cementitious material filling slurries gradually decrease with the increase in mass concentration. Taking a c/t ratio of 1:3 as an example, when the mass concentration increases from 64% to 70%, the slump of the cement and cementation powder filling slurries decreases by 11% and 12%, respectively, and the spread decreases by 38% and 36%, respectively. The slump and spread of the slurry also gradually decrease with the decrease in the c/t ratio. Taking a mass concentration of 68% as an example, when the c/t ratio decreases from 1:2 to 1:15, the slump of the cement and cementation powder filling slurries decreases by 2.9% and 3.8%, respectively, and the spread decreases by 28% and 26%, respectively.

As the slurry concentration increases and the c/t ratio decreases, the flowability of the slurry gradually decreases. With finer particle size, increasing the slurry concentration results in more particles, which increases friction between particles and thus affects the slurry’s flowability. As the c/t ratio increases, under a fixed water amount, adding more cement and reducing tailings is equivalent to releasing part of the water that was originally physically adsorbed and encapsulated by the tailings, thereby relatively increasing the free water content in the filling slurry. Free water acts as the medium for slurry flow, and an increase in its content significantly improves flowability.

### 3.2. Analysis of Rheological Results

**Figure 3:**
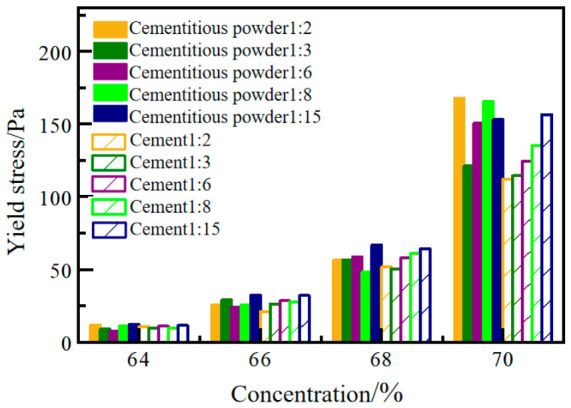
The yield stress of the slurry filled with two different cementitious materials.

Based on the test and summary analysis of the rheological parameters of filler slurries with cement and solidifying powder as the two cementitious materials, a comparative analysis of the yield stress of the filler slurries with the two cementitious materials is presented. The results are shown in Figure 3.

Under various c/t ratios, the yield stress gradually increases with the rise in slurry mass concentration, with a relatively gentle increase in the 64–68% range and a more noticeable increase in the 68–70% range, especially for c/t ratios of 1:8 and 1:15, mainly due to the reduction in the c/t ratio. Lowering the c/t ratio makes the filling slurry more dilute, reducing the interparticle friction and increasing the slurry’s fluidity. Once the slurry exceeds the critical concentration, its fluidity deteriorates significantly, leading to a marked increase in yield stress.

Under the same mix conditions, except for a few cases, the yield stress of cementation powder filling slurry is lower than that of cement filling slurry, indicating that under the same conditions, cementation powder filling slurry has better fluidity than cement filling slurry. Compared to cement, cementation powder enhances the internal hydration reaction within the filling slurry, producing finer and smoother hydration products that act as a lubricant between tailings particles. This reduces direct contact and internal friction among the solid particles, making the entire particle system easier to slide during shearing, thereby lowering the yield stress of the filling slurry and exhibiting good fluidity.

### 3.3. Analysis of Bleeding Rate and Shrinkage Rate

The calculation formulas for bleeding rate and shrinkage rate are shown in Equation (4) and Equation (5), respectively.

(4) B=VwWGGw×100%

(5) $$\delta =\frac{V_{1}}{V_{2}}\times 100\%$$

where *B* is the bleeding rate, %; *V_W_* is the total mass of bleeding water, g; *W* is the water content of the slurry, g; *G* is the total mass of slurry plus container, g; *G*_W_ is the mass of slurry, g; *δ* is the shrinkage rate of the slurry, %; *V*_1_ is the reduced volume after shrinkage, mL; *V*_2_ is the total volume of the original slurry, mL.

**Figure 4:**
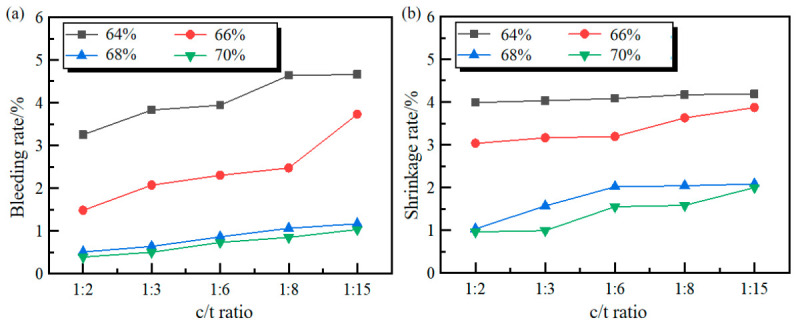
The bleeding and shrinkage results of cement filling slurry: (**a**) bleeding rate; (**b**) shrinkage rate.

**Figure 5:**
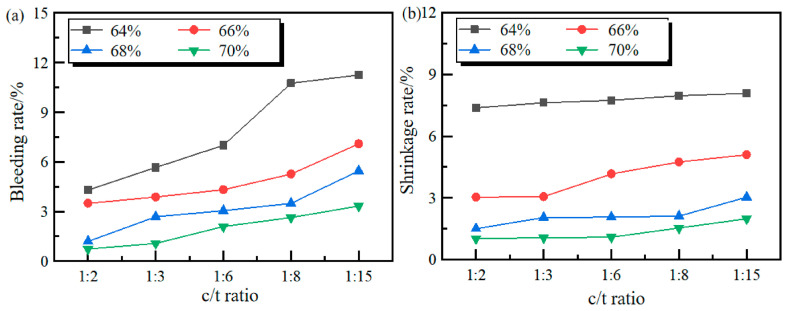
The bleeding and shrinkage results of cementation powder filling slurry: (**a**) bleeding rate; (**b**) shrinkage rate.

By comparing Figure 4 and Figure 5, it can be seen that for both cement and cementation powder as cementing materials, the bleeding rate and shrinkage rate of the filling slurry gradually decrease with the increase in slurry mass concentration. Similarly, the bleeding rate and shrinkage rate also gradually decrease with the increase in the c/t ratio, indicating that the stability of the slurry for both cementing materials gradually improves with the increase in mass concentration and c/t ratio. Comparing the bleeding and shrinkage test results of cement and cementation powder, it can be seen that under the same conditions, the bleeding rate and shrinkage rate of cement filling slurry are lower than those of the cementation powder. The experimental results show that in terms of bleeding and shrinkage performance, cement is superior to cementation powder.

As the c/t ratio increases, because cement particles are extremely fine, increasing the amount of cement fills the voids between coarse particles, making the packing of solid particles more compact, reducing the connected pores within the system and increasing the tortuosity. Water molecules find it difficult to find continuous, unobstructed paths to migrate upwards and thus adhere to fine pores, resulting in a decrease in the bleeding rate.

## 4. Analysis of Backfill Strength Characteristics

### 4.1. Effect of Cementing Material Type on Backfill Strength

**Figure 6:**
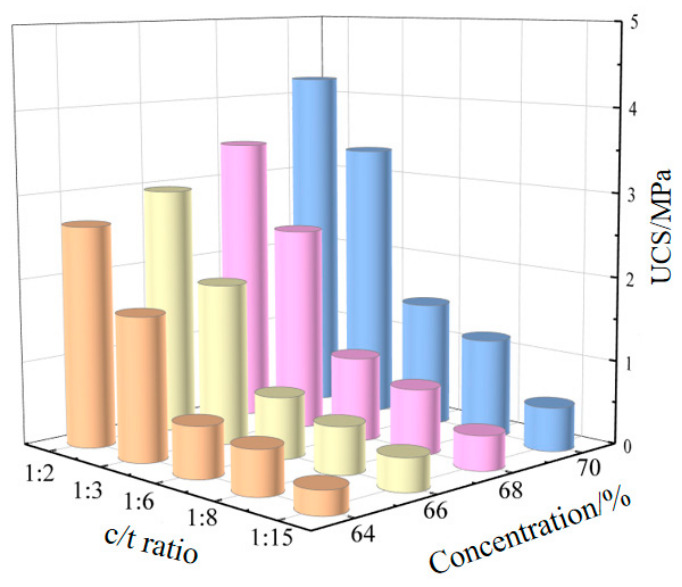
The UCS of cemented fillers varying with the c/t ratio.

At a curing age of 28 days, the UCS of cemented fillers and cementation powder fillers indicates that within the tested range, the strength of cementation powder filler is generally higher than that of cemented fill [34]. Analysis of Figure 6 shows that when the c/t ratio increases from 1:15 to 1:2, the strength range of cemented fillers at slurry concentrations of 64%, 66%, 68%, and 70% is 0.28 MPa~2.65 MPa, 0.39 MPa~2.96 MPa, 0.41 MPa~3.44 MPa, and 0.52 MPa~4.21 MPa, respectively. Under the same c/t ratio and slurry concentration, the strength of cementation powder filler is higher than that of cemented filler by 7.14%~19.25%, 5.13%~21.96%, 30.23%~31.71%, and 30.64%~42.31% respectively.

Taking a mass concentration of 70% as an example, under a gray sand ratio of 1:2~1:15, the 28-day strength of the cementation powder is 1.31, 1.13, 1.64, 1.39, and 1.43 times that of cement, respectively. Compared with cement, the cementation powder shows superior strength characteristics in the filler. The cementation powder is generally finer than cement, has a larger specific surface area, and is more easily dispersed in the filling slurry, fully encapsulating the tailings particles. At the same time, the cementation powder contains more active substances, and after dissolving in water, it exhibits better cohesiveness with the entire tailings. Greater external force is required when the fill experiences deformation and failure.

### 4.2. The Effect of the Cement-to-Sand Ratio on the Strength of the Backfill

**Figure 7:**
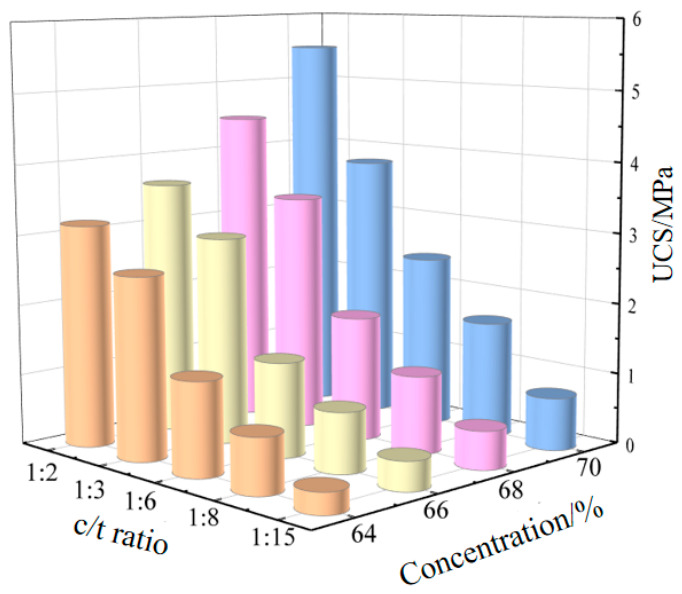
The UCS of cementation powder fillers varying with the c/t ratio.

The UCS of cemented filler and cementation powder filler varying with the c/t ratio is shown in Figure 6 and Figure 7, respectively. It can be seen from the figures that, under the same conditions, the strength of fillers increases as the c/t ratio increases.

Taking the strength of the cementation powder filler as an example, the strength increase trend is as follows: using a c/t ratio of 1:15 as the baseline, when the ratio is increased to 1:8, the strength rises by 1.04 to 1.60 times; when the ratio is increased to 1:6, the strength rises by 2.3 to 3.4 times; when the ratio is increased to 1:3, the strength rises by 4.1 to 7.6 times; and when the ratio is increased to 1:2, the strength rises by 6.4 to 9.5 times.

Analyzing the strength of the filler shows that increasing the c/t ratio can effectively enhance the strength of the filler. Increasing the c/t ratio directly increases the thickness and coverage of the cement paste on the tailings sand particles. The hydration products (such as C-S-H) generated after the hydration of cementitious materials encapsulate and bridge the tailings sand particles, forming a three-dimensional network skeleton. The higher the c/t ratio, the richer the gel phase in a unit volume, increasing the number of bonding points and surfaces on the tailings sand particles, resulting in a denser structure and reduced porosity. This enhances the interparticle bonding force, allowing the backfill to transmit stress more effectively under external forces, which is macroscopically manifested as a significant increase in compressive strength.

### 4.3. Effect of Slurry Concentration on the Strength of the Backfill

**Figure 8:**
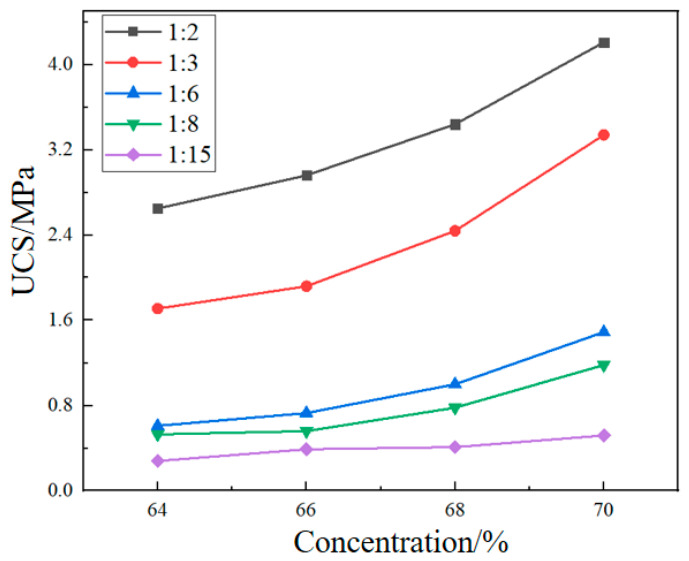
The strength variation of cemented fillers with concentration.

**Figure 9:**
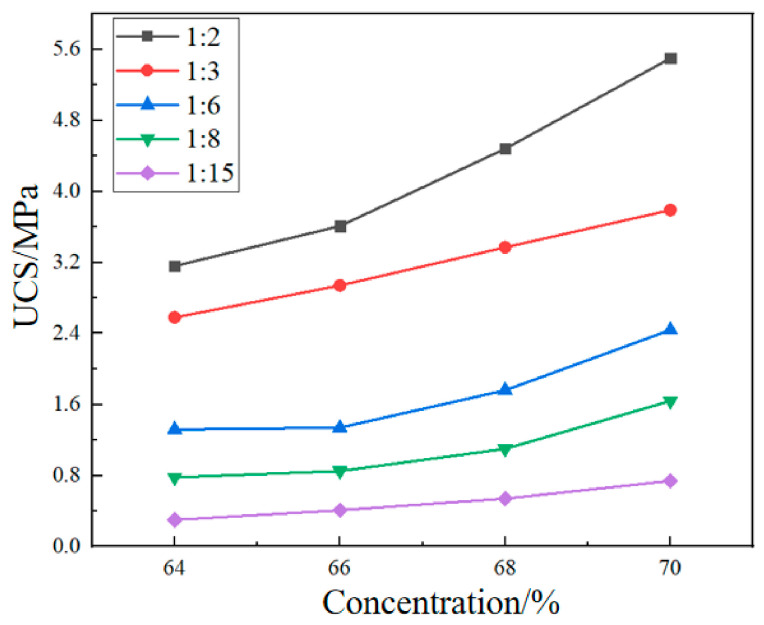
The strength variation of cementation powder fillers with concentration.

Figure 8 and Figure 9 show the strength variation of cemented filler and cementation powder filler with concentration. Under different c/t ratios, both the strength of the cemented filler and the cementation powder filler increased with the increase in slurry concentration, and the growth rate increased with the increase in the c/t ratio. When the mass concentration of cemented filler was increased from 64% to 70%, the strength of the filler increased by 1.59, 1.95, 2.44, 2.23, and 1.86 times for c/t ratios of 1:2, 1:3, 1:6, 1:8, and 1:15, respectively. The strength increase in the cementation powder filler ranged from 0.47 to 1.47 times.

As the concentration of the filling slurry increases, the content of tailings and cementitious solid particles per unit volume also increases, while the proportion of water decreases. The distance between solid particles is shortened due to increased density, which facilitates efficient bridging by hydration products between adjacent particles, forming a dense microstructure. At the same time, the reduction in free water content in the slurry can significantly reduce voids and capillary channels formed due to water evaporation, thereby lowering internal defects of the backfill. Higher-concentration slurries also possess suitable fluidity, which can inhibit tailings segregation and bleeding, ensuring uniform distribution of solid particles and promoting the complete development of the cementitious network.

## 5. Analysis of the Strength Enhancement Mechanism of Fillers

Laboratory tests are typically conducted under controlled conditions, whereas industrial-scale operations are subject to various variable factors that may weaken backfill strength. Specifically, fluctuations in raw material quality (such as variations in tailings gradation and cement activity), insufficient mixing homogeneity, setting or segregation caused by delays between mixing and placement, and suboptimal curing conditions (e.g., low temperature or inadequate humidity) can all hinder the cement hydration process, thereby affecting the development of fillers’ strength.

### 5.1. Analysis of the Internal Factors of Tailings Affecting Filler Strength

The strength of the filler is largely influenced by internal factors such as its material composition, microstructure, and internal defects. As the main aggregate of the filler, tailings’ physical, chemical, and mineralogical properties directly determine the microstructure and macroscopic performance of the filler. Serving as both the framework and active component of the filler, the particle size, mineral composition, and chemical characteristics of tailings influence the porosity, interfere with the hydration reaction, and affect the interfacial bonding and overall compactness of the filler, thereby comprehensively constraining its ultimate strength. By analyzing the basic physical and chemical properties of whole tailings, the main internal factors affecting filler strength include the following two aspects:

(1) High content of fine particles in tailings: Based on previous particle size distribution analyses, it was found that the content of particles smaller than 200 mesh exceeds 80%, and the content of ultrafine particles smaller than 600 mesh is close to 60%. The high proportion of fine particles, combined with a lack of coarse particles and poor continuity in particle size distribution, results in overall low filler strength.
(2) Tailings contain F and P chemical components and clay minerals such as kaolinite and montmorillonite. F^−^ and PO_4_^3−^ ions can react with Ca^2+^ in the cement paste to form insoluble precipitates of calcium fluoride (CaF_2_) or calcium phosphate (Ca_3_(PO_4_)_2_). These precipitates coat the surface of cement particles, forming a physical barrier that hinders the contact between water molecules and the cementitious materials (especially C_3_A and C_3_S), thereby affecting the mechanical properties of the backfill. Soluble F and P hinder cement hydration, making it difficult for the backfill to set and harden, and posing the risk of expansion and cracking. Clay minerals reduce the fluidity of the filling slurry, complicate concentration increases, and make dewatering at the slurry collection site difficult. These combined factors negatively affect the filler strength.

### 5.2. Microstructure Analysis

Factors affecting the strength of the filler, in addition to the high content of fine tailings particles and the complex chemical composition of the tailings, are also related to the type of cementing material. This was confirmed by early mix ratio tests using cementation powder and cement. In this study, XRD, SEM, and other micro-testing methods were used to analyze the factors influencing filler strength with different cementing materials.

#### 5.2.1. SEM Analysis

**Figure 10:**
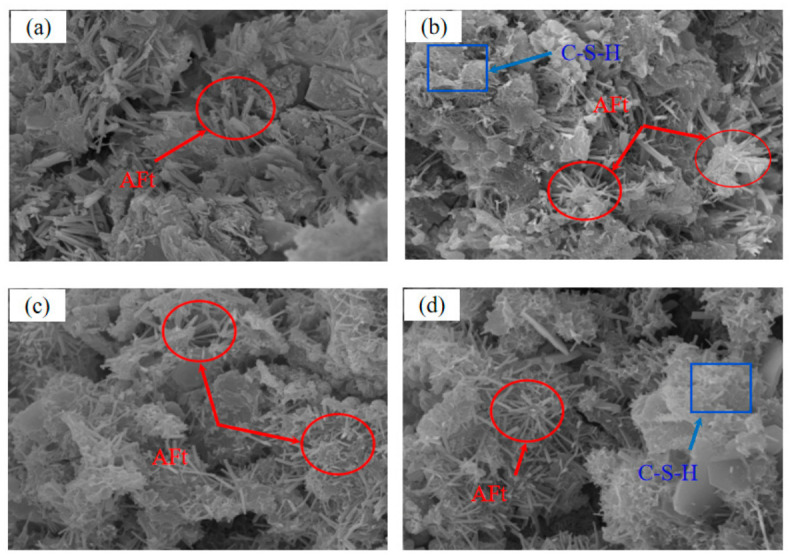
The microstructure of fillers: (**a**,**b**) cementation powder and (**c**,**d**) cement.

A scanning electron microscope was used to observe the microstructure of the cementation powder and the cement filler, and the results are shown in Figure 10.

Both the cementation powder fillers and the cemented fillers increase in the main hydration products such as ettringite and C-S-H gel as the curing age increases, and their microstructure gradually becomes denser, which is also the reason for the strength growth. The hydration products of the cementation powder filler mainly include C-S-H gel, while those of the cemented filler mainly include ettringite, C-S-H gel, portlandite, and hydrotalcite. At the same time, compared with the cemented filler, the cementation powder filler contains more C-S-H gel and coarser ettringite, resulting in higher filler strength; additionally, in the cement products, the platy portlandite and layered hydrotalcite somewhat weaken the structure and internal framework of the filler, which is also one of the reasons that affects the strength of the cemented filler.

#### 5.2.2. XRD Analysis

**Figure 11:**
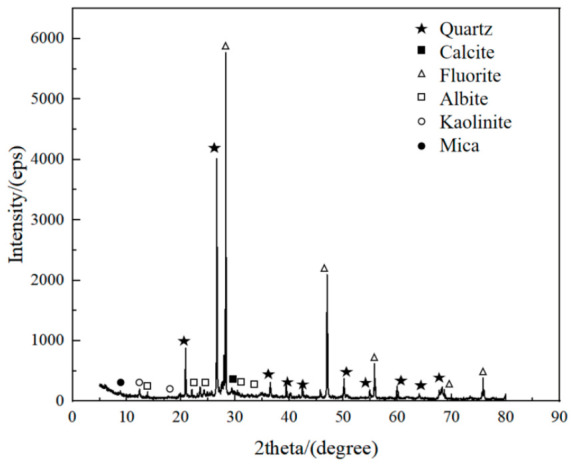
The results of phase composition of cementation powder fillers (28 d).

**Figure 12:**
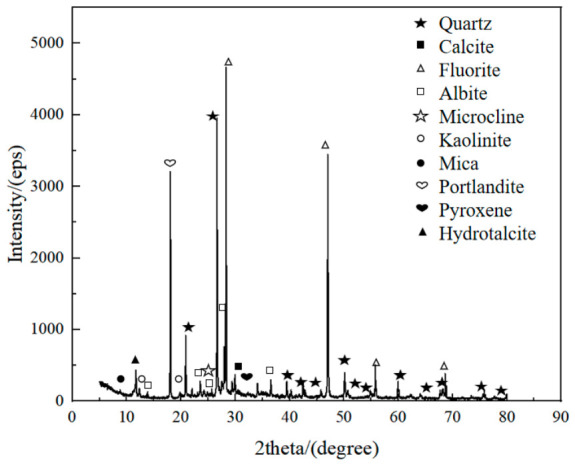
The results of phase composition of cemented fillers (28 d).

The XRD analysis results of the cementation powder filler and the cemented filler are shown in Figure 11 and Figure 12, respectively. Analysis of the spectra indicates that there are significant differences in the hydration products of the cementation powder and cemented fillers. The hydration products of the cementation powder filler mainly include quartz, fluorite, feldspar, kaolinite, mica, and calcite. In contrast, the hydration products of the cemented filler are more complex, mainly including quartz, fluorite, feldspar, kaolinite, mica, portlandite, gypsum, calcite, pyroxene, and hydrotalcite.

Comparing the types of hydration products between the cementation powder and cemented fillers, it can be seen that the cemented filler has more complex hydration products. Compared with the cementation powder filler, the cemented filler produces additional portlandite, pyroxene, and hydrotalcite. The presence of gypsum in the cemented filler indicates an excess of gypsum in the hydration system, which affects the formation of early hydration gels and, in turn, influences the strength of the filler. The formation of hydrotalcite in the cemented filler, a layered anionic clay mineral, has certain negative effects on the structural stability of the filler.

### 5.3. Analysis of the Hydration Mechanism of the Backfill Body

The hydration mechanism of the filler is the core chemical process for its strength development. This process is primarily dominated by the reaction between cementation materials (usually cement) and water, and it also involves the physicochemical effects of aggregates such as tailings to jointly form the hardened structure.

The hydration reaction begins when the clinker minerals of the cementitious material (mainly tricalcium silicate (C_3_S), dicalcium silicate (C_2_S), tricalcium aluminate (C_3_A), and tetracalcium aluminoferrite (C_4_AF)) come into contact with water. C_3_S and C_2_S hydrolyze rapidly, producing C-S-H gel and calcium hydroxide (CH). C-S-H gel is the primary source of strength in the filler. It has an amorphous colloidal structure with a large specific surface area, allowing it to interweave and encapsulate tailings particles to form a cohesive three-dimensional network. At the same time, the generated CH provides an alkaline environment that promotes the continuation of the reaction and helps activate potential reactive components in the tailings to undergo secondary pozzolanic reactions, generating additional C-S-H gel, thereby enhancing later strength and density.

The hydration rate of the aluminate phases (C_3_A, C_4_AF) is relatively fast. They react with gypsum regulators to form crystal products such as ettringite. These crystals fill the pores in the early stages and interweave with the C-S-H gel, further reinforcing the microstructure. The entire hydration process is accompanied by the release of hydration heat and an increase in solid volume, gradually transforming the initially fluid slurry into a dense hardened body.

During this process, tailings not only serve as a physical skeleton, but their surface characteristics and mineral composition also affect the nucleation and growth of hydration products, as well as the structure and strength of the interfacial transition zone.

## 6. Conclusions

In this paper, the physical and chemical properties of the tailings are examined, and the influence of two cementitious materials on the flow performance of the filling slurry and the strength of the filling body is systematically compared from a combined analysis of the structural characteristics of the tailings from the combined aspects, and the strength formation mechanism of the CTB is revealed on this basis. The main conclusions are as follows:

(1) Under the same conditions, cementation powder filling slurry has better fluidity than cement filling slurry.
(2) According to the laser particle size test results, the tailings are graded well, but the continuity is unstable and coarse particles are lacking. The tailings contained high contents of clay minerals such as kaolinite and F and P, which had an adverse effect on filler strength.
(3) The bleeding rate and shrinkage rate of cemented filling slurry were smaller than those of cementation powder, and the experimental results showed that cement was better than cementation powder in terms of bleeding and sedimentation properties. Under the same conditions, the slump and diffusion of cementation powder filling slurry were better than those of cement, and the working characteristics of cemented powder filling slurry were better. At the same time, the fluidity of the cementation powder filling slurry is better than that of the cement filling slurry.
(4) When the c/t ratio and slurry concentration are the same, the strength of the cementation powder filling slurry at each age is significantly higher than that of cement. When the filling slurry concentration was 70%, the 28-day strength of the cementation powder fillers was 1.31, 1.13, 1.64, 1.39, and 1.43 times that of the cemented fillers under c/t ratios ranging from 1:2 to 1:15, respectively.
(5) From the analysis of the microstructure characteristics of the filler, the cementation powder filler has more C-S-H gel than the hydration product of the cement filler, and the calcite is thicker, which makes the filler stronger. In addition, the plate-like calcium hydroxide stone and layered hydrotalcite in the cement products have a certain degree of weakening the structure and internal skeleton of the filler, which is also one of the reasons affecting the strength of the cemented filler.

## Figures and Tables

**Table 1:** The physical properties of tailings.

Physical Properties	Density/(t·m^−3^)	Loose Density/(t·m^−3^)	Porosity/%
Tailing	2.533	1.121	55.740

**Table 2:** The primary chemical composition of materials.

Chemical Composition (Concentration/%)	Al_2_O_3_	CaO	SiO_2_	K_2_O	MgO	TiO
Tailings	7.24	12.29	58.17	3.21	0.44	0.48
Cement	4.13	64.78	22.33	0.12	3.21	/
Cementation powder	3.58	57.27	24.68	0.48	4.68	/
Chemical Composition (Concentration/%)	Na_2_O	P_2_O_5_	S	Fe	F	/
Tailings	1.97	0.19	0.05	3.11	7.46	/
Cement	0.13	0.03	0.05	4.71	/	/
Cementation powder	1.96	0.79	5.01	1.32	/	/

## Data Availability

The raw data supporting the conclusions of this article will be made available by the authors on request.
